# Genetically Determined Circulating Lactase/Phlorizin Hydrolase Concentrations and Risk of Colorectal Cancer: A Two-Sample Mendelian Randomization Study

**DOI:** 10.3390/nu16060808

**Published:** 2024-03-12

**Authors:** Sihao Han, Jiemin Yao, Hajime Yamazaki, Samantha A. Streicher, Jianyu Rao, Roch A. Nianogo, Zuofeng Zhang, Brian Z. Huang

**Affiliations:** 1 Department of Epidemiology, Fielding School of Public Health, University of California, Los Angeles, CA 90095, USA; jieminyao@g.ucla.edu (J.Y.); jrao@mednet.ucla.edu (J.R.); niaroch@ucla.edu (R.A.N.); zfzhang@ucla.edu (Z.Z.); 2 Section of Clinical Epidemiology, Department of Community Medicine, Graduate School of Medicine, Kyoto University, Kyoto 606-8303, Japan; yamazaki.hajime.7n@kyoto-u.ac.jp; 3 Center for Innovative Research for Communities and Clinical Excellence (CiRC2LE), Fukushima Medical University, Fukushima 960-1295, Japan; 4 Cancer Epidemiology Division, Population Sciences in the Pacific Program, University of Hawaii Cancer Center, University of Hawaii at Manoa, Honolulu, HI 96813, USA; sstreicher@cc.hawaii.edu; 5 Department of Pathology and Laboratory Medicine, David Geffen School of Medicine, University of California, Los Angeles, Los Angeles, CA 90095, USA; 6 Norris Comprehensive Cancer Center, University of Southern California, Los Angeles, CA 90033, USA; brian.huang@usc.edu; 7 Department of Population and Public Health Sciences, Keck School of Medicine, University of Southern California, Los Angeles, CA 90033, USA

**Keywords:** colorectal cancer, lactase/phlorizin hydrolase, lactose non-persistence, milk digestion, Mendelian randomization

## Abstract

Previous research has found that milk is associated with a decreased risk of colorectal cancer (CRC). However, it is unclear whether the milk digestion by the enzyme lactase-phlorizin hydrolase (LPH) plays a role in CRC susceptibility. Our study aims to investigate the direct causal relationship of CRC risk with LPH levels by applying a two-sample Mendelian Randomization (MR) strategy. Genetic instruments for LPH were derived from the Fenland Study, and CRC-associated summary statistics for these instruments were extracted from the FinnGen Study, PLCO Atlas Project, and Pan-UK Biobank. Primary MR analyses focused on a *cis*-variant (rs4988235) for LPH levels, with results integrated via meta-analysis. MR analyses using all variants were also undertaken. This analytical approach was further extended to assess CRC subtypes (colon and rectal). Meta-analysis across the three datasets illustrated an inverse association between genetically predicted LPH levels and CRC risk (OR: 0.92 [95% CI, 0.89–0.95]). Subtype analyses revealed associations of elevated LPH levels with reduced risks for both colon (OR: 0.92 [95% CI, 0.89–0.96]) and rectal cancer (OR: 0.92 [95% CI, 0.87, 0.98]). Consistency was observed across varied analytical methods and datasets. Further exploration is warranted to unveil the underlying mechanisms and validate LPH’s potential role in CRC prevention.

## 1. Introduction

Colorectal cancer (CRC) is one of the most common forms of cancer in the digestive system. There are estimated to be over 1.9 million incident cases and 93,500 CRC-related deaths in 2020, making CRC the third most common cancer and the second leading cause of cancer-related death worldwide [1]. The complex etiology of CRC points to a confluence of genetic, dietary, and lifestyle determinants of risk [2,3,4].

Lactase-phlorizin hydrolase (LPH) is a pivotal enzyme in the human body that helps hydrolyze lactose, the main carbohydrate in milk, into glucose and galactose [5,6]. The reduced expression or activity of LPH, known as lactase non-persistence (LNP), leads to a clinical condition called lactose intolerance, in which milk and other dairy products cannot be properly digested. Individuals with lactose intolerance experience symptoms such as abdominal pain, bloating, diarrhea, nausea, and vomiting after consumption of milk and other dairy products [5,6]. Genetically, LPH is encoded by the lactase gene (LCT) on chromosome 2. Genetic expression of LCT has been found to be regulated by single nucleotide polymorphisms (SNPs) located on the gene MCM6, a regulatory region 14 kb upstream from the LCT gene [6,7,8]. Specifically, the SNP rs4988235 on MCM6 confers the LNP phenotype.

Diminished LPH levels or activity, leading to lactose maldigestion, are linked to decreased calcium [9] and vitamin D intake [10], along with a reduced abundance of beneficial gut bacteria, Bifidobacterium [11]. Observational studies have reported that reduced calcium [12,13] and vitamin D [14,15] intake are associated with increased CRC risk, suggesting protective roles of calcium and vitamin D in CRC development. In addition, clinical studies have shown that dietary intake of Bifidobacterium modulates gut microbiota towards CRC prevention [16]. Given LPH’s pivotal role in milk digestion and its downstream influence on crucial nutrient absorption and gut microbiota composition, it may also have a significant impact on CRC susceptibility. In addition, LPH could potentially serve as a potential candidate biomarker for CRC risk stratification or a druggable target for CRC treatment, as several other circulating proteins associated with CRC risk have been implemented for these purposes [17,18,19,20]. Yet, the specific role of LPH in the development of CRC remains unclear, highlighting the need for detailed studies exploring this potential association.

There has been no research directly studying the relationship between LPH levels and CRC risk in the medical literature. Instead, previous epidemiologic studies have investigated this relationship using LNP status, LPH-related SNPs, and dietary milk intake as proxies for LPH levels [21,22,23,24,25,26,27]. However, these studies have several limitations, including exposure misclassification, residual confounding, and reverse causality. For instance, lactase persistence/non-persistence status was often binarily defined by individual genotype. However, the negative impacts of lactose maldigestion among lactase-non-persistent individuals are actually determined by continuous residual LPH expression levels [6,28,29]. In addition, CRC patients undergoing adjuvant 5-fluorouracil chemotherapy can develop secondary lactose intolerance due to gastrointestinal damage [30,31], disrupting small intestine enzyme and transporter functions [32]. Consequently, the potential for reverse causation (i.e., CRC leading to reduced LPH levels and thus milk intake) remains plausible.

To circumvent these challenges, we utilize Mendelian Randomization (MR) analysis, an innovative method that employs genetic variants as instrumental variables (IVs) for LPH levels [33]. The random assignment of these variants during meiosis helps mitigate confounding bias and reverse causality issues, offering a robust means to explore potential causality [33,34,35]. While conventional genome-wide MR studies encompass both *cis*-variants (i.e., located near the gene of interest) and *trans*-variants (i.e., often located on different chromosomes), there is a rising trend in *cis*-MR studies that exclusively use *cis*-variants as IVs, especially in contexts where protein expression is a key consideration [36,37,38,39,40]. The appeal of *cis*-MR studies has grown due to their potential for drug target identification and validation [38,40]. In our study, we focus on continuous LPH levels as the exposure, selecting both *cis*- and *trans*-variants associated with LPH levels from a large-scale genome-wide association study (GWAS). We then use sets of (1) only *cis*-variants and (2) combined *cis*- and *trans*-variants as separate IVs in our MR analyses.

This study leverages MR to probe the potential causal influence of genetically determined elevated LPH levels on the risk of CRC and its subtypes, namely colon and rectal cancer. Utilizing publicly accessible summary-level GWAS data from three large-scale, independent cohorts of European ancestry, we seek to enhance our understanding of the genetic underpinnings of CRC and inform future preventive strategies.

## 2. Materials and Methods

### 2.1. Study Design

Our study utilized a two-sample MR approach, using genetic variants as IVs, to investigate whether there is a causal relationship between elevated LPH levels and the risk of CRC. The MR analyses rest on three fundamental assumptions: (1) the Relevance assumption establishes that the genetic IVs are associated with the exposure (e.g., LPH levels); (2) the Independence assumption states that the genetic IVs have no correlation with potential confounders; and (3) the Exclusion restriction assumption dictates that the genetic IVs could only affect the outcome of interest (e.g., CRC) via the exposure (i.e., no horizontal pleiotropy where genetic IVs can affect multiple outcomes) [41].

**Figure 1:**
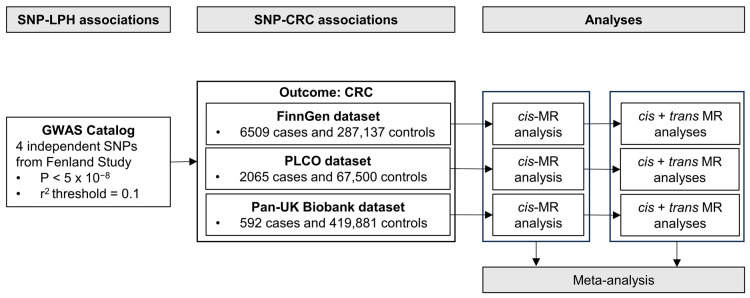
Schematic overview of the study design for the primary Mendelian Randomization analyses. Abbreviations: SNP, single nucleotide polymorphism; LPH, lactase-phlorizin hydrolase; CRC, colorectal cancer; GWAS, genome-wide association study; MR, Mendelian Randomization.

The schematic overview of our study design is presented in Figure 1. Our process commenced with the selection of genetic instruments for LPH levels from the GWAS Catalog [42], followed by the extraction of summary statistics of these selected genetic instruments from prior GWAS of CRC risk performed in three independent cohorts: the FinnGen Study, the Prostate, Lung, Colorectal, and Ovarian Cancer Screening Trial (PLCO) Atlas Project, and the Pan-UK Biobank. Each cohort had prior ethical approvals, negating the need for additional approvals for this study.

To assess the causal effect of elevated LPH levels on CRC risk, we primarily conducted two-sample MR analyses in each cohort using a *cis*-variant for LPH levels. The results from the three cohorts were subsequently integrated using meta-analysis. For validation, MR analyses incorporating all variants (*cis*- + *trans*-) were also performed. Further, this identical workflow was used for the analysis of CRC subtypes (i.e., colon and rectal cancer). Our study followed the Strengthening the Reporting of Observational Studies in Epidemiology Using Mendelian Randomization (STROBE-MR) reporting guidelines [43].

### 2.2. Genetic Instruments

Genetic instruments for LPH levels were retrieved from the NHGRI-EBI GWAS Catalog, a large-scale open GWAS database collaboratively developed by the European Bioinformatics Institute (EBI) and the Human Genome Research Institute (NHGRI) with over 24,000 traits (www.ebi.ac.uk/gwas, accessed on 2 May 2023) [42]. Our focus was on the Fenland Study data from the GWAS Catalog, which offers the largest and most recent GWAS of LPH levels (GWAS Catalog accession ID: GCST90248315). Summaries of the study are listed in Table 1. The Fenland Study consisted of 10,708 genotyped participants of European ancestry who were recruited from general practice surgeries in the Cambridgeshire region of the UK from 2005 to 2015 [44]. Genotyping was conducted using three different arrays (Affymetrix UK Biobank Axiom array [Affymetrix, Santa Clara, CA, USA], Illumina Infinium Core Exome 24v1 [Illumina, San Diego, CA, USA], and Affymetrix SNP5.0 [Affymetrix, Santa Clara, CA, USA]), and levels for each protein target were measured using the rank-based inverse normal-transformed aptamer abundance method [44]. GWAS analysis was then performed using the transformed protein levels, with the residuals used as input for the genetic association analyses [45]. The beta coefficients for each protein target, representing one standard deviation (SD) change in normalized plasma abundance of protein per effect allele of the SNPs, were estimated, adjusting for age, sex, sample collection site, and the first ten principal components [44].

Our study selected SNPs associated with LPH levels at the genome-wide significant threshold of *p* < 5 × 10^−8^ [46]. Correlated SNPs were excluded according to measures of linkage disequilibrium (LD) r^2^ < 0.1 and minor allele frequency (MAF) > 0.01 based on the European populations from the 1000 Genomes phase 3 reference panel using the SNPclip online tool (https://ldlink.nih.gov/, accessed on 4 May 2023) [47].

Following exclusions, our analysis included four variants, one *cis*-variant (rs4988235) and three *trans*-variants (rs516246, rs532436, and rs641476), that were used as genetic instruments to genetically predict LPH levels. The characteristics of the genetic instruments for elevated LPH levels included in our study are presented in Table 2. Four independent SNPs associated with *MCM6* (rs4988235), *FUT2* (rs516246), *ABO* (rs532436), and *GAREM1* (rs641476) were selected based on the genome-wide significance level (*p* < 5 × 10^−8^) and LD-based pruning (r^2^ < 0.1). Overall, the four selected SNPs accounted for 36.42% of the observed variance in elevated LPH levels, with the *cis*-variant rs4988235 contributing the majority of the variance.

To assess the strength of the genetic instruments selected, we calculated R^2^ (the percent variation in LPH levels explained by the genetic instrument) and the Cragg–Donald *F*-statistics (the strength of the association between the genetic instrument and LPH levels) for each LPH-associated SNP using the formula: R^2^ = β^2^ × 2 × EAF × (1 − EAF) and F = R^2^ × (N − 2)/(1 − R^2^), where EAF denotes the effect allele frequency of the SNP and N represents the sample size of the exposure GWAS [48,49]. A *F*-statistic greater than 10 indicates strong genetic instruments for the MR analyses [50]. The F-statistics for the four SNPs ranged from 87.01 to 5340.06, underscoring their strength as genetic instruments for MR analyses.

### 2.3. Outcome Data Sources

A summary of the GWAS datasets for CRC is presented in Table 1. Summary-level data pertaining to the association of SNPs with CRC were obtained from three publicly available GWAS: (1) the FinnGen Study (available at https://www.finngen.fi/en/access_results, accessed on 2 May 2023); (2) the PLCO Atlas Project (available at https://exploregwas.cancer.gov/plco-atlas/#/gwas/summary, accessed on 2 May 2023); and (3) the Pan-UK Biobank (available at https://pan.ukbb.broadinstitute.org/, accessed on 2 May 2023). Detailed information for these studies was reported in the original publications [51,52,53]. CRC cases were identified by: (1) ICD-10 codes C18–C20 in the FinnGen Study; (2) ICD-O-2 codes 180, 182–189, 199, 209, 212, and 218 in the PLCO Atlas; and (3) self-report through verbal interview with a trained nurse in the Pan-UK Biobank [51,52,53]. To minimize population stratification bias, only GWAS results from individuals of European ancestry were included.

All genetic association estimates between the SNPs and CRC were calculated using logistic regression comparing cases and controls, adjusting for age, sex, and genetic principal components (the first ten in the FinnGen consortium and Pan-UK Biobank, and the first twenty in the PLCO Atlas). In addition, some studies also included study-relevant covariates in their logistic regression models, such as age^2^ (in the Pan-UK Biobank), study center (in the PLCO Atlas), and genotyping batch (in the FinnGen Study).

We extracted estimates (e.g., effective alleles, beta coefficients, standard errors, and p-values) for the associations between the selected genetic instruments and the risk of CRC and CRC subtypes (colon and rectal cancer) from the FinnGen, PLCO Atlas, and Pan-UK Biobank GWAS. For SNPs not available in these GWAS, we identified proxy SNPs in linkage disequilibrium (r^2^ > 0.7 within a ±500,000 base pairs window) based on the European populations from the 1000 Genomes phase 3 reference panel utilizing the LDProxy online tool (https://ldlink.nih.gov/, accessed on 4 May 2023) [47]. All four genetic instruments were found in the PLCO and Pan-UK Biobank datasets. Rs532436 was not available in the FinnGen dataset, and thus we used the proxy SNP rs635634, which was in high linkage disequilibrium with rs532436 (r^2^ = 0.99). Details of the genetic association between the SNPs and the risk of CRC are presented in Table 3.

### 2.4. Statistical Power Calculation

The statistical power of our MR analyses was calculated using an online tool (https://sb452.shinyapps.io/power/, accessed on 15 May 2023) with several parameters, including the total sample size, the percent variance in the exposure explained by the genetic instruments (R^2^), and the ratio of cases to controls [54]. Calculations were performed separately for each cohort. The significance level for the power calculation was set at α = 0.05. Results from the power calculation indicated that our study has an 80% power to detect a 6% change in the odds of CRC per SD increase in normalized plasma LPH levels.

### 2.5. Statistical Analysis

Effect alleles were defined for each SNP as the allele contributing to increased LPH levels. We performed strand alignment to harmonize the relationships between genetic instruments and CRC, as well as between LPH levels and CRC for the same allele. We primarily performed the Wald ratio two-sample *cis*-MR using rs4988235 as the genetic instrument. For validation, we then employed the inverse-variance weighted (IVW) two-sample MR across all four genetic instruments. The IVW method assumes that all SNPs are valid instruments and that horizontal pleiotropic effects are absent or balanced, constraining the intercepts to zero [55]. The Cochran’s Q statistic and I^2^ index were used to test for the presence of heterogeneity, which is an indicator of whether the IVW estimates on LPH levels and CRC risk are different across different genetic variants [56].

Further enhancing the robustness of our investigation, we performed a series of sensitivity MR analyses, including penalized IVW, robust IVW, penalized robust IVW, MR–Egger, weighted median, mode-based estimation, and MR–Lasso. The robust IVW method uses robust regression to downweight outliers, while the penalized IVW method improves the robustness of the estimates by penalizing the weights of genetic instruments with heterogeneous causal estimates for the outcome [57,58]. The penalized robust IVW method further provides robustness both to outliers and to data points with high leverage through robust regression [57]. The MR–Egger method allows the inclusion of horizontal pleiotropic SNPs and provides a bias-corrected exposure-outcome effect estimate, with a deviating intercept indicating mean pleiotropic effects [59]. Despite relaxing the exclusion restriction assumption, MR–Egger mandates the InSIDE (Instrument Strength Independent of Direct Effect) assumption, which requires that the associations of the genetic instruments with the exposure and the direct effects of the genetic instruments on the outcome are independent [60]. Consequently, we also incorporated MR analyses that do not require the InSIDE assumption (e.g., weighted median and the mode-based estimation) [59,61]. To assess the distortions of the IVW estimate from any heterogeneity or horizontal pleiotropy, MR–Lasso was used to detect and remove pleiotropic outliers [62].

The effect estimates of genetically predicted LPH on CRC and its subtypes were reported as odds ratios (ORs), along with their 95% confidence intervals (CIs), per one SD increase in normalized plasma abundance of LPH. Each SNP’s association was plotted against its corresponding effect on CRC risk. To evaluate the potential influence of a single SNP on MR results, iterative leave-one-out analyses were executed [60].

All of the primary and sensitivity MR analyses were conducted separately within each of the three outcome data sources (i.e., FinnGenn, PLCO Atlas, and Pan-UK Biobank). For comparison and consolidation of effect estimates from varying data sources, we utilized meta-analysis with fixed effects models to integrate the IVW estimates across the three cohorts. The degree of heterogeneity between the IVW estimates was quantified using the I^2^ index and Cochran Q statistics [63].

All statistical tests were two-sided, with the level of significance predetermined at *p* < 0.05. We performed all analyses using R version 4.1.2 (The R Foundation for Statistical Computing) [64]. We used the “MendelianRandomization” package [65] for MR analyses and the “meta” package for meta-analyses [66].

## 3. Results

### 3.1. FinnGen Dataset

The FinnGen GWAS summary statistics on CRC consisted of 6509 CRC cases and 287,137 controls. Using only the *cis*-variant rs4988235 as the genetic instrument, the FinnGen dataset showed that genetically determined higher levels of LPH were associated with decreased odds of CRC (OR per SD higher normalized plasma abundance of LPH: 0.91 [95% CI, 0.88–0.95], *p* < 0.001) (Table S1). The IVW estimate from the MR analysis using all LPH-associated genetic variants showed similar results as the *cis*-MR analysis (OR: 0.92 [95% CI, 0.88–0.95], *p* < 0.001) (Table S1, Figure S1A). Results for sensitivity analyses were presented in Table S1 and Figures S1–S3. Little heterogeneity across SNPs was evidenced by Cochran’s Q statistics (Q = 2.5, *p* = 0.482), and sensitivity analyses produced consistent results. There was no evidence of horizontal pleiotropy according to the MR–Egger results (P_Egger-intercept_ = 0.552). Based on the leave-one-out analysis (Figure S3A), the primary influence on the effect came from the SNP rs4988235 on *MCM6*, which is the most well-characterized SNP responsible for LPH synthesis and the only *cis*-variant selected in the GWAS for LPH levels [4,6].

### 3.2. PLCO Dataset

The PLCO GWAS dataset included 2065 CRC participants and 67,500 controls. The PLCO dataset illustrated a non-significant association between genetically determined elevated LPH levels and CRC risk in the *cis*-MR (OR: 0.92 [95% CI, 0.85–1.00], *p* = 0.063) (Table S1). Similar results were found in the MR analysis including all genetic instruments (OR: 0.94 [95% CI, 0.85–1.03], *p* = 0.170), whereas the confidence interval was slightly wider than that in the *cis*-MR (Table S1, Figure S1B). Table S1 and Figures S1–S3 show the results from the sensitivity analyses. With penalized robust IVW, the association became significant (OR: 0.94 [95% CI, 0.90–0.98], *p* = 0.002), indicating the presence of potential outliers. Results from the MR–Egger, weighted median, and mode-based estimation analyses did not provide strong evidence for horizontal pleiotropic effects among the SNPs (Table S1). The leave-one-out analysis plot suggested that the MR IVW estimates were largely influenced by rs4988235, which was consistent with results in the FinnGen dataset (Figure S3B).

### 3.3. Pan-UK Biobank Dataset

There were 592 CRC cases and 419,881 controls in the Pan-UK Biobank. The *cis*-MR Wald ratio did not provide evidence supporting the effect of genetically determined elevated LPH levels on CRC risk in the Pan-UK Biobank dataset (OR: 1.00 [95% CI, 0.87–1.14], *p* = 0.971), and this result was similar with the IVW estimate including both *cis*- and *trans*-variants (OR: 1.03 [95% CI, 0.83–1.27], *p* = 0.812) (Table S1, Figure S1C). In addition, the intercept for the MR–Egger analysis was not significantly different from zero (P_Egger-intercept_ = 0.712), indicating little evidence of horizontal pleiotropic effects in the selected genetic instruments. Sensitivity analyses mirrored the IVW estimate, with the leave-one-out analysis affirming rs4988235’s substantial impact (Figure S3C).

### 3.4. Meta-Analysis Combining FinnGen, PLCO, and Pan-UK Biobank Results

**Figure 2:**
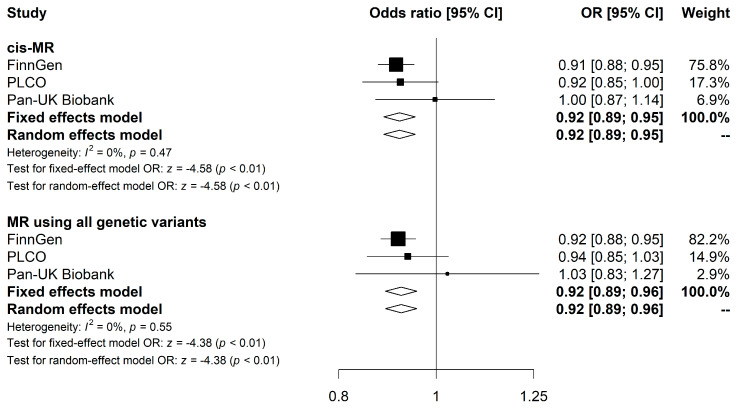
Meta-analysis results for the association of LPH levels with CRC risk using MR analyses. Forest plots show results from *cis*-MR and MR using all genetic variants. Squares represent study-specific MR estimates. Diamonds represent meta-analyzed MR estimates using fixed and random effects models. Detailed results from the MR analyses and sensitivities analyses for each CRC GWAS study are presented in Table S1. Abbreviations: LPH, lactase-phlorizin hydrolase; CRC, colorectal cancer; MR, Mendelian Randomization.

Meta-analysis combining the *cis*-MR estimates from FinnGen, PLCO, and Pan-UK Biobank showed an inverse association between genetically predicted elevated LPH and CRC risk (OR: 0.92 [95% CI, 0.89–0.95], *p* < 0.001), with no discernible heterogeneity in the effect across the three datasets (I^2^ = 0%, P_cochran-Q_ = 0.470) (Figure 2). Similarly, the combined IVW estimate for MR studies utilizing all four genetic variants showed a sightly attenuated association (OR: 0.93 [95% CI, 0.89–0.96], *p* < 0.001). We did not find strong evidence indicating heterogeneity across the three datasets (I^2^ = 0%, P_cochran-Q_ = 0.554) (Figure 2).

### 3.5. CRC Subtype-Specific MR Analyses

CRC subtype-specific MR analyses are reported in Tables S2–S4, and sensitivity analysis results are presented in Figures S4–S6 and S8–S10. The *cis*-MR analysis of the FinnGen dataset revealed an inverse association between genetically predicted elevated LPH levels and colon cancer risk (OR: 0.92 [95% CI, 0.87–0.97], *p* = 0.001, Table S2). Although similar Wald ratio estimates were observed in the PLCO and Pan-UK Biobank datasets, statistical significance was not reached, potentially due to small sample sizes (PLCO: OR 0.93 [95% CI, 0.85–1.02], *p* = 0.144; Pan-UK Biobank: OR 0.95 [95% CI, 0.86–1.05], *p* = 0.285, Table S2). Combining the *cis*-MR results from the three datasets, the meta-analyzed estimate (Table S4, Figure S7) suggested a significant association between genetically predicted higher LPH levels and decreased risk of colon cancer (OR: 0.92 [95% CI, 0.89–0.96], *p* < 0.001). Results from the MR analyses utilizing all four genetic instruments further confirmed the association with similar estimates but wider confidence intervals (meta-analyzed OR: 0.93 [95% CI, 0.89–0.97], *p* < 0.001) (Tables S2 and S4, Figure S7).

With respect to rectal cancer, the FinnGen dataset indicated an inverse association between genetically predicted elevated LPH levels and rectal cancer susceptibility when using the single *cis*-variant rs4988235 (OR: 0.91 [95% CI, 0.85–0.97], *p* = 0.005, Table S3). The PLCO dataset suggested a negative but non-significant estimate (OR: 0.86 [95% CI, 0.70–1.06], *p* = 0.172, Table S3). Results from the Pan-UK Biobank dataset, however, demonstrated an incongruous positive, albeit non-significant, estimate (OR: 1.13 [95% CI, 0.91–1.40], *p* = 0.267, Table S3). The subsequent meta-analysis (Table S4, Figure S11) suggested an inverse association between elevated LPH levels and rectal cancer risk (OR: 0.92 [95% CI, 0.87, 0.98], *p* = 0.0083), and moderate heterogeneity was observed across the datasets (I^2^ = 50%, P_cochran-Q_ = 0.136). Further MR analyses including both *cis*- and *trans*-variants showed consistent results (Tables S3 and S4, Figure S11), indicating the robustness of our *cis*-MR estimates.

## 4. Discussion

In this study, we leveraged summary-level statistics from three large-scale GWAS of European ancestry and employed a two-sample MR framework to investigate the potential causal relationship between LPH levels and CRC risk using both *cis*-variants and all genetic instruments (*cis-* + *trans-*). The results from the *cis*-MR analysis provided genetic evidence suggesting an inverse causal association between elevated LPH levels and CRC risk. This finding was consistent and validated by MR analyses using both *cis-* and *trans*-variants. Further MR analyses by CRC subtypes indicated that this causal relationship seemed applicable to both colon cancer and rectal cancer.

While the FinnGen dataset showed a significant inverse association between genetically predicted elevated LPH levels and CRC risk, the findings from the PLCO and Pan-UK Biobank datasets were not statistically significant, likely due to insufficient statistical power attributed to smaller sample sizes and lower case-to-control ratios. We confirmed this hypothesis through power calculations, revealing 85% power in the FinnGen dataset to detect a 6% change in the odds of CRC, compared with just 39% and 15% power in the PLCO and Pan-UK Biobank datasets, respectively. Therefore, to bolster statistical power, we conducted a meta-analysis of the separate MR analyses within each of the three cohorts. Subgroup analyses for colon and rectal cancer revealed similar trends. With a relatively small number of rectal cancer cases in both the PLCO (320 cases) and Pan-UK Biobank (301 cases) datasets, these analyses were likely hindered by limited statistical power.

It is worth noting that the Pan-UK Biobank dataset showed a higher number of colon cancer cases compared to overall CRC cases. This discrepancy might be explained by the case identification method in the Pan-UK Biobank, which is reliant on self-reported cancer diagnoses and therefore subject to potential measurement error. Although more accurate cancer case ascertainment methods might be employed in individual-level UK Biobank datasets, such information was not available in the publicly accessible summary statistic data that we utilized.

The potential underlying biological mechanisms linking elevated LPH levels with reduced CRC risk warrant further exploration. Evidence suggests that lactase persistent individuals typically consume more milk than their lactase-non-persistent counterparts [67,68]. Given the known impact of milk consumption on CRC risk [26,27,69,70,71,72,73,74], it is plausible that the protective effect of LPH on CRC risk is partially mediated through catalyzed products of milk [75,76,77] and key milk components, namely calcium [12,13,78] and vitamin D [14,79]. Other milk-derived compounds, such as butyric acid, conjugated linoleic acid, sphingolipids, and lactoferrin [80,81,82], also contribute to the protective effect of LPH. Moreover, the effect of LPH on gut microbiota diversity could also play a role in modifying CRC risk [11].

Calcium and vitamin D, abundant components of milk, have been recognized for their multifaceted roles in CRC prevention. Calcium’s protective effects can be attributed to its capacity to bind secondary bile acids and ionized fatty acids, thereby reducing their toxicity on colonocytes and inhibiting mucosal proliferation [6]. In addition, it may activate certain signaling pathways via the calcium-sensing receptor (CaSR), including E-cadherin expression promotion, beta-catenin/T cell factor activation suppression, and p38 mitogen-activated protein kinase cascade activation [78]. There is also evidence linking calcium to a lower risk of mutations in the *KRAS* gene, a significant determinant in the carcinogenesis of CRC [6]. Vitamin D modulates molecular pathways relevant to CRC development, including the downregulation of the *COX-2* gene and the upregulation of 15-hydroxyprostaglandin dehydrogenase (15-PDGH), leading to a reduction in local prostaglandin levels and hence inhibiting cancer cell survival [14]. Moreover, it interferes with β-catenin-mediated gene transcription, primarily by promoting Vitamin D receptor (VDR) binding to β-catenin, emphasizing its suppressive role on tumor growth [79].

Other milk compounds, such as butyric acid, conjugated linoleic acid, and lactoferrin, may also contribute to CRC prevention [80,81,82]. These components have shown various anti-carcinogenic effects in in vitro and animal studies, ranging from suppressing proliferation to enhancing immune function [80,81,82,83,84,85]. Additionally, LPH levels might impact CRC risk by modifying the gut microbiota. For instance, studies have linked increased LPH levels to a greater abundance of *Bifidobacterium* [11], which is known for augmenting antitumor immunity and facilitating the efficacy of immunotherapy [86]. In this context, our MR findings provide genetic support for this biological rationale, underscoring the relevance of LPH metabolism in CRC prevention.

While no study has directly investigated the effects of LPH on CRC risk, our findings are comparable to prior epidemiologic studies investigating CRC risk associated with LNP status or genetic instruments for milk consumption. Two studies conducted in Finnish and Hungarian populations observed a statistically significant increased risk of CRC risk among LNP individuals, with ORs reported at 1.40 and 4.04, respectively [22,24]. Although other studies conducted in British, Spanish, and Italian populations observed no association between LNP and CRC, these had limited statistical power due to small sample sizes (44-283 CRC cases) [22,25]. Furthermore, two other studies using rs4988235 as a genetic instrument for milk consumption found that genetically predicted milk intake was associated with a reduced risk of CRC (reported ORs of 0.89 and 0.95) [26,27]. This is similar to the effect size observed in our current analysis for genetically predicted LPH levels and CRC risk (OR 0.92) using the same *cis*-variant (rs4988235).

Our findings on the protective effect of LPH against CRC development highlight its potential role in CRC prevention and treatment. Specifically, LNP individuals identified through screening methods, such as lactose breath tests or genetic testing of the rs4988235 polymorphism, could benefit from specific dietary recommendations (e.g., calcium or vitamin D supplements) to mitigate CRC risk. Such targeted interventions could not only enhance individual health outcomes, but also contribute to more personalized and potentially cost-effective approaches to CRC risk management. Furthermore, LPH can perhaps serve as a novel therapeutic target for CRC, providing potential avenues for CRC treatment strategies.

Our study has several notable strengths. We implemented a *cis*-MR approach as our primary analysis, which not only mitigates biases such as residual confounding and reverse causation that typically complicate observational studies, but also minimizes potential horizontal pleiotropy. The use of the *cis*-variant (rs4988235), located within the *MCM6* gene and in close proximity of the LPH-encoded gene *LCT*, ensures that the observed effects on CRC can be attributed solely to variations in LPH expression, given the regulatory role of rs4988235 [6,7,8]. This study’s findings suggest the potential therapeutic role of LPH for CRC, underscoring its clinical significance. Furthermore, the utilization of all genetic variants (*cis-* + *trans-*) served as a validation of the *cis*-MR approach and allowed for a series of sensitivity analyses. These included various MR methods, such as weighted median, mode-based estimation, and MR–Egger, which helped to examine the potential effects of horizontal pleiotropy from selected genetic instruments. Previous studies may have also been subject to several limitations, such as binary definitions of lactase persistence status and potential violations of the relevance assumption of MR [21,22,23,24,25,26,27]. Our study addressed these issues by using genetically predicted continuous LPH levels as the exposure and selecting genetic instruments directly associated with LPH levels from large-scale GWAS datasets. By our calculations, the SNPs selected in our study explained 36.43% of the variance in LPH levels, with rs4988235 displaying a strong association with LPH levels (variance explained: 33.28%). In addition, by using distinct GWAS datasets for LPH levels (exposure) and CRC (outcome) in our two sample MR analyses, we also reduced the potential inflation of bias associated with weak instrument variables [87]. Furthermore, we accounted for heterogeneity introduced by specific SNPs with outlier causal estimates by employing penalized IVW and MR–Lasso estimations. The application of leave-one-out analyses also helped us verify the consistency of estimates across genetic instruments and determine whether specific SNPs substantially influenced our causal estimates. We further integrated three large-scale, independent GWAS datasets into our MR analyses and meta-analyses, ensuring sufficient sample sizes for the outcome. Lastly, by conducting MR analyses across different CRC subtypes, we offered a comprehensive view of LPH’s potential biological role in various tumor locations.

However, our study has some limitations. We acknowledge that the limited number of CRC cases in the Pan-UK Biobank, and especially the smaller number of rectal cancer cases across all three cohorts, could have constrained our study’s statistical power. To mitigate this limitation, we employed meta-analysis techniques, maximizing data utilization to yield more robust results and inferences. A further limitation of our study lies in our exclusive inclusion of individuals of European descent. It is worth noting that the prevalence of lactase non-persistence significantly varies across populations; it is highest in East Asians (for example, 85% in Chinese and 100% in South Koreans) and lowest in individuals of Northern European descent (for instance, 8% in Finns and 7.8% in Swedes) [8]. Consequently, these variations in LPH levels among different populations restrict the generalizability of our findings to individuals of European ancestry. Future research should include other populations and delve into sex-specific causal estimates for a more nuanced understanding of LPH and CRC.

This study, to our knowledge, is the first to explore the causal relationships between LPH levels and the risk of CRC using MR analyses with large-scale GWAS datasets. The findings underscore the importance of LPH and its downstream effects in influencing CRC risk. Moreover, it may provide new insights into preventive strategies and a potential drug target for interventions aimed at reducing the burden of CRC. Further studies are necessary to better delineate these mechanisms and validate the potential of LPH as a biomarker for CRC risk.

## 5. Conclusions

Our study suggests that there is an inverse causal relationship between LPH levels and CRC risk. These findings, consistent across cohorts for both colon and rectal cancers, highlight a potential causal role for LPH as a preventative biomarker. Further study is needed to clarify the mechanisms and extend these findings to other populations.

## Figures and Tables

**Table 1:** Summary of GWAS datasets used for LPH levels and CRC.

LPH GWAS					CRC GWAS						Colon Cancer GWAS		Rectal Cancer GWAS	
Study	First Author (Year)	Sample Size	Population	Sex	Study	N Cases	N Controls	Population	Sex	CRC Ascertainment	N Cases	N Controls	N Cases	N Controls
Fenland Study	Pietzner (2021) [44]	10,708	100% European	53% Female	FinnGen Study	6509	287,137	100% European	42% Female	ICD10: C18–C20	3935	287,137	2361	287,137
					PLCO Atlas	2065	67,500	100% European	45% Female	ICD-O-2 Site: 180/182–189/199/209/212/218	1611	65,142	320	65,142
					Pan-UK Biobank	592	419,881	100% European	44% Female	Self-reported diagnosis of large bowel cancer/colorectal caner	1384	419,089	301	420,172

Abbreviations: GWAS: genome-wide association studies; LPH: lactase-phlorizin hydrolase; CRC: colorectal cancer.

**Table 2:** Characteristics of genetic instruments for elevated LPH levels from the GWAS identified in the GWAS Catalog.

RSID	Position (GRCh38)	Effect Allele	Other Allele	EAF	R^2^	F	Beta	SE	*p*-Value	Associated Gene	*cis*/*trans* Variant for LPH Levels
rs4988235	chr2:135851076	A	G	0.31	33.28%	5340.06	0.882	0.011	3 × 10^−1451^	*MCM6*	*cis*
rs516246	chr19:48702915	C	T	0.49	0.81%	87.01	0.127	0.013	2 × 10^−22^	*FUT2*	*trans*
rs532436	chr9:133274414	G	A	0.20	1.27%	137.41	0.199	0.016	3 × 10^−35^	*ABO*	*trans*
rs641476	chr18:32225445	C	T	0.61	1.07%	115.85	0.150	0.013	5 × 10^−30^	*GAREM1*	*trans*

Abbreviations: LPH, lactase-phlorizin hydrolase; GWAS: genome-wide association studies; EAF: effect allele frequency; SE: standard error.

**Table 3:** Summary of four genetic instruments and their proxies (where necessary) from the FinnGen, PLCO, and Pan-UK Biobank GWAS on CRC.

SNP Selected	Effect Allele	Beta	SE	*p*-Value
FinnGen				
rs4988235	A	−0.081	0.020	0.001
rs516246	C	−0.024	0.020	0.291
rs635634 (proxy) ^a^	G	0.006	0.025	0.386
rs641476	C	0.004	0.020	0.967
PLCO				
rs4988235	A	−0.072	0.039	0.063
rs516246	C	−0.034	0.032	0.292
rs532436	G	0.047	0.040	0.237
rs641476	C	0.020	0.033	0.537
Pan-UK Biobank				
rs4988235	A	−0.002	0.061	0.971
rs516246	C	−0.072	0.051	0.162
rs532436	G	0.144	0.066	0.030
rs641476	C	0.061	0.053	0.245

^a^ rs635634 at chr9:133279427 (effect allele G) was used as a proxy for rs532436 (r^2^ = 0.99) in the FinnGen study. Abbreviations: CRC: colorectal cancer; GWAS: Genome-wide association studies; SNP: single nucleotide polymorphism; SE: standard error.

## Data Availability

Publicly available datasets were analyzed in this study. The GWAS summary statistics for LPH levels (the GWAS Catalog) are available at https://www.ebi.ac.uk/gwas/studies/GCST90248315 (accessed on 2 May 2023). The GWAS summary statistics for CRC are available at https://www.finngen.fi/en/access_results (accessed on 2 May 2023) for the FinnGen Study, https://exploregwas.cancer.gov/plco-atlas/#/ (accessed on 2 May 2023) for the PLCO Atlas, and https://pan.ukbb.broadinstitute.org/downloads/index.html (accessed on 2 May 2023) for the Pan-UK Biobank.

## Supplementary Materials

The following supporting information can be downloaded at: https://www.mdpi.com/article/10.3390/nu16060808/s1. Table S1. Associations of genetically predicted elevated LPH Levels and CRC in the FinnGen, PLCO, and Pan-UK Biobank datasets; Table S2. Associations of genetically predicted elevated LPH levels and colon cancer in the FinnGen, PLCO, and Pan-UK Biobank datasets; Table S3. Associations of genetically predicted elevated LPH levels and rectal cancer in the FinnGen, PLCO, and Pan-UK Biobank datasets; Table S4. Meta-analysis results for the association between elevated LPH levels and CRC, colon cancer, and rectal cancer; Figure S1. Scatter plots of the IVW and MR–Egger methods investigating the effect of elevated LPH levels on CRC in the FinnGen, PLCO, and Pan-UK Biobank datasets; Figure S2. Forest plots of the IVW estimates on the association between genetically predicted LPH levels and CRC risk for each genetic instrument in the FinnGen, PLCO, and Pan-UK Biobank datasets; Figure S3. Leave-one-out analyses for the MR analysis on LPH levels and CRC risk in the FinnGen, PLCO, and Pan-UK Biobank datasets; Figure S4. Scatter plots of the IVW and MR–Egger methods investigating the effect of elevated LPH levels on colon cancer in the FinnGen, PLCO, and Pan-UK Biobank datasets; Figure S5. Forest plots of the IVW estimates on the association between genetically predicted LPH levels and colon cancer risk for each genetic instrument in the FinnGen, PLCO, and Pan-UK Biobank datasets; Figure S6. Leave-one-out analyses for the MR analysis on LPH levels and colon cancer risk in the FinnGen, PLCO, and Pan-UK Biobank datasets; Figure S7. Meta-analysis results for the association of elevated LPH levels with colon cancer risk using *cis*-MR and MR using all genetic variants; Figure S8. Scatter plots of the IVW and MR-Egger methods investigating the effect of elevated LPH levels on rectal cancer in the FinnGen, PLCO, and Pan-UK Biobank datasets; Figure S9. Forest plots of the IVW estimate on the association between genetically predicted LPH levels and rectal cancer risk for each genetic instrument in the FinnGen, PLCO, and Pan-UK Biobank datasets; Figure S10. Leave-one-out analyses for the MR analysis on LPH levels and rectal cancer risk in the FinnGen, PLCO, and Pan-UK Biobank datasets; Figure S11. Meta-analysis results for the association of elevated LPH levels with rectal cancer risk using *cis*-MR and MR using all genetic variants.

## Abbreviations

CRC:	Colorectal cancer
LPH:	Lactase-phlorizin hydrolase
LNP:	Lactase non-persistence
SNP:	Single nucleotide polymorphism
MR:	Mendelian Randomization
IV:	Instrumental variable
GWAS:	Genome-wide association study
PLCO:	the Prostate, Lung, Colorectal, and Ovarian Cancer Screening Trial
STROBE-MR:	Strengthening the Reporting of Observational Studies in Epidemiology Using Mendelian Randomization
EBI:	European Bioinformatics Institute
NHGRI:	Human Genome Research Institute
SD:	Standard deviation
LD:	Linkage disequilibrium
MAF:	Minor allele frequency
EAF:	Effect allele frequency
IVW:	Inverse-variance weighted
InSIDE:	Instrument Strength Independent of Direct Effect
OR:	Odds ratio
CI:	Confidence interval
CaSR:	Calcium-sensing receptor
15-PDGH:	15-hydroxyprostaglandin dehydrogenase
VDR:	Vitamin D receptor
