# Supplementary material for: Genetically Determined Circulating Lactase/Phlorizin Hydrolase Concentrations and Risk of Colorectal Cancer: A Two-Sample Mendelian Randomization Study
Source: Nutrients. 2024 Mar 12;16(6):808. doi: 10.3390/nu16060808 (PMC10975724; doi:10.3390/nu16060808)
Supplement: Supplementary file 1 [file nutrients-16-00808-s001.zip › nutrients-2825526-supplementary.pdf]

Tables and Figures

Supplementary Tables

Table S1. Associations of genetically predicted elevated LPH Levels and CRC in the FinnGen, PLCO, and Pan-UK Biobank datasets. 3

Table S2. Associations of genetically predicted elevated LPH levels and colon cancer in the FinnGen, PLCO, and Pan-UK Biobank datasets. 4

Table S3. Associations of genetically predicted elevated LPH levels and rectal cancer in the FinnGen, PLCO, and Pan-UK Biobank datasets. 5

Table S4. Meta-analysis[a] results for the association between elevated LPH levels and CRC, colon cancer, and rectal cancer. 6

Supplementary Figures

Figure S1. Scatter plots of the IVW and MR-Egger methods investigating the effect of elevated LPH levels on CRC in the FinnGen, PLCO, and Pan-UK Biobank datasets....... 7

Figure S2. Forest plots of the IVW estimates on the association between genetically predicted LPH levels and CRC risk for each genetic instrument in the FinnGen, PLCO, and Pan-UK Biobank datasets. 8

Figure S3. Leave-one-out analyses for the MR analysis on LPH levels and CRC risk in the FinnGen, PLCO, and Pan-UK Biobank datasets. 9

Figure S4. Scatter plots of the IVW and MR-Egger methods investigating the effect of elevated LPH levels on colon cancer in the FinnGen, PLCO, and Pan-UK Biobank datasets.. 10

Figure S5. Forest plots of the IVW estimates on the association between genetically predicted LPH levels and colon cancer risk for each genetic instrument in the FinnGen, PLCO, and Pan-UK Biobank datasets. 11

Figure S6. Leave-one-out analyses for the MR analysis on LPH levels and colon cancer risk in the FinnGen, PLCO, and Pan-UK Biobank datasets. 12

Figure S7. Meta-analysis results for the association of elevated LPH levels with colon cancer risk using *cis*-MR and MR using all genetic variants.. 13

Figure S8. Scatter plots of the IVW and MR-Egger methods investigating the effect of elevated LPH levels on rectal cancer in the FinnGen, PLCO, and Pan-UK Biobank datasets. 14

Figure S9. Forest plots of the IVW estimate on the association between genetically predicted LPH levels and rectal cancer risk for each genetic instrument in the FinnGen, PLCO, and Pan-UK Biobank datasets. 15

Figure S10. Leave-one-out analyses for the MR analysis on LPH levels and rectal cancer risk in the FinnGen, PLCO, and Pan-UK Biobank datasets. 16

Figure S11. Meta-analysis results for the association of elevated LPH levels with rectal cancer risk using *cis*-MR and MR using all genetic variants.. 17

Table S1. Associations of genetically predicted elevated LPH Levels and CRC in the FinnGen, PLCO, and Pan-UK Biobank datasets.

Method	OR	Lower 95% CI	Upper 95% CI	P-value	MR-Egger Intercept	Heterogeneity: Q, P
FinnGen						
***cis*-MR**						
Wald ratio	0.91	0.88	0.95	1.3×10^{-5}		
**MR using all genetic variants**						
IVW	0.92	0.88	0.95	1.8×10^{-5}		
Penalized IVW	0.92	0.87	0.97	0.001		
Robust IVW	0.92	0.90	0.93	8.9×10^{-37}		
Penalized robust IVW	0.92	0.90	0.94	2.9×10^{-15}		
MR-Egger	0.90	0.85	0.96	0.001	0.009, 0.552	2.5, 0.482
Weighted median	0.92	0.88	0.95	3.3×10^{-5}		
Mode-based estimation	0.91	0.88	0.95	1.6×10^{-5}		
MR Lasso	0.92	0.88	0.95	1.8×10^{-5}		
PLCO						
***cis*-MR**						
Wald ratio	0.92	0.85	1.00	0.0631		
**MR using all genetic variants**						
IVW	0.94	0.85	1.03	0.170		
Penalized IVW	0.94	0.85	1.03	0.170		
Robust IVW	0.94	0.90	0.98	0.002		
Penalized robust IVW	0.94	0.90	0.98	0.002		
MR-Egger	0.91	0.78	1.05	0.193	0.020, 0.532	3.9, 0.273
Weighted median	0.93	0.86	1.02	0.109		
Mode-based estimation	0.92	0.85	1.01	0.070		
MR Lasso	0.94	0.85	1.03	0.170		
Pan-UK Biobank						
***cis*-MR**						
Wald ratio	1.00	0.87	1.14	0.9707		
**MR using all genetic variants**						
IVW	1.03	0.83	1.27	0.812		
Penalized IVW	1.07	0.77	1.49	0.681		
Robust IVW	1.02	0.94	1.11	0.569		
Penalized robust IVW	1.07	0.88	1.30	0.510		
MR-Egger	0.98	0.69	1.40	0.901	0.029, 0.712	7.9, 0.049
Weighted median	1.02	0.89	1.17	0.787		
Mode-based estimation	1.01	0.88	1.16	0.925		
MR-Lasso	1.03	0.83	1.27	0.812		

LPH, lactase-phlorizin hydrolase; CRC, colorectal cancer; OR: odds ratio; CI: confidence interval; MR: Mendelian Randomization; IVW: inverse variance weighted.

Table S2. Associations of genetically predicted elevated LPH levels and colon cancer in the FinnGen, PLCO, and Pan-UK Biobank datasets.

Method	OR	Lower 95% CI	Upper 95% CI	P-value	MR-Egger Intercept	Heterogeneity: Q, P
FinnGen						
***cis*-MR**						
Wald ratio	0.92	0.87	0.97	0.0011		
**MR using all genetic variants**						
IVW	0.92	0.87	0.97	0.002		
Penalized IVW	0.92	0.87	0.97	0.002		
Robust IVW	0.92	0.91	0.93	5×10^{-31}		
Penalized robust IVW	0.92	0.91	0.93	5×10^{-31}		
MR-Egger	0.92	0.83	1.01	0.074	0.002, 0.641	3.3, 0.345
Weighted median	0.93	0.89	0.97	0.002		
Mode-based estimation	0.92	0.87	0.97	0.001		
MR Lasso	0.92	0.87	0.97	0.002		
PLCO						
***cis*-MR**						
Wald ratio	0.93	0.85	1.02	0.1435		
**MR using all genetic variants**						
IVW	0.95	0.85	1.06	0.361		
Penalized IVW	0.98	0.82	1.16	0.814		
Robust IVW	0.95	0.90	1.00	0.038		
Penalized robust IVW	0.98	0.87	1.10	0.708		
MR-Egger	0.91	0.76	1.09	0.311	0.024, 0.534	4.5, 0.211
Weighted median	0.95	0.86	1.04	0.253		
Mode-based estimation	0.94	0.85	1.03	0.170		
MR Lasso	0.95	0.85	1.06	0.361		
Pan-UK Biobank						
***cis*-MR**						
Wald ratio	0.95	0.86	1.05	0.2851		
**MR using all genetic variants**						
IVW	0.95	0.84	1.07	0.401		
Penalized IVW	0.95	0.84	1.07	0.401		
Robust IVW	0.95	0.91	0.98	0.006		
Penalized robust IVW	0.95	0.91	0.98	0.006		
MR-Egger	0.94	0.76	1.16	0.564	0.006, 0.099	4.8, 0.184
Weighted median	0.94	0.85	1.04	0.234		
Mode-based estimation	0.94	0.85	1.05	0.233		
MR Lasso	0.95	0.84	1.07	0.401		

LPH, lactase-phlorizin hydrolase; OR: odds ratio; CI: confidence interval; MR: Mendelian Randomization; IVW: inverse variance weighted.

Table S3. Associations of genetically predicted elevated LPH levels and rectal cancer in the FinnGen, PLCO, and Pan-UK Biobank datasets.

Method	OR	Lower 95% CI	Upper 95% CI	P-value	MR-Egger Intercept	Heterogeneity: Q, P
FinnGen						
***cis*-MR**						
Wald ratio	0.91	0.85	0.97	0.0051		
**MR using all genetic variants**						
IVW	0.92	0.86	0.98	0.009		
Penalized IVW	0.92	0.85	0.99	0.035		
Robust IVW	0.92	0.89	0.94	1×10^{-11}		
Penalized robust IVW	0.92	0.89	0.95	3×10^{-6}		
MR-Egger	0.89	0.81	0.97	0.013	0.023, 0.328	1.6, 0.654
Weighted median	0.91	0.85	0.97	0.006		
Mode-based estimation	0.91	0.85	0.97	0.005		
MR Lasso	0.92	0.86	0.98	0.009		
PLCO						
***cis*-MR**						
Wald ratio	0.86	0.70	1.06	0.1720		
**MR using all genetic variants**						
IVW	0.86	0.70	1.05	0.86		
Penalized IVW	0.86	0.70	1.05	0.86		
Robust IVW	0.86	0.82	0.90	0.86		
Penalized robust IVW	0.86	0.82	0.90	0.86		
MR-Egger	0.88	0.66	1.18	0.88	-0.017, 0.780	4.5, 0.211
Weighted median	0.86	0.70	1.06	0.151		
Mode-based estimation	0.86	0.70	1.05	0.135		
MR Lasso	0.86	0.69	1.07	0.173		
Pan-UK Biobank						
***cis*-MR**						
Wald ratio	1.13	0.91	1.40	0.2670		
**MR using all genetic variants**						
IVW	1.10	0.90	1.35	0.360		
Penalized IVW	1.10	0.90	1.35	0.360		
Robust IVW	1.10	1.03	1.18	0.006		
Penalized robust IVW	1.10	1.03	1.18	0.006		
MR-Egger	1.22	0.91	1.64	0.178	-0.064, 0.323	2.1, 0.559
Weighted median	0.91	0.85	0.97	0.315		
Mode-based estimation	1.13	0.91	1.41	0.270		
MR Lasso	1.10	0.90	1.35	0.360		

LPH, lactase-phlorizin hydrolase; OR: odds ratio; CI: confidence interval; MR: Mendelian Randomization; IVW: inverse variance weighted.

Table S4. Meta-analysis[a] results for the association between elevated LPH levels and CRC, colon cancer, and rectal cancer.

	OR	Lower 95% CI	Upper 95% CI	P-value	I^2	Q-statistics, P
CRC						
***cis*-MR**						
Fixed effects model	0.92	0.89	0.95	4.66×10^{-6}	0%	1.51, 0.4695
Random effects model	0.92	0.89	0.95	4.66×10^{-6}		
**MR using all variants**						
Fixed effects model	0.92	0.89	0.96	1.20×10^{-5}	0%	1.18, 0.5536
Random effects model	0.92	0.89	0.96	1.20×10^{-5}		
Colon cancer						
***cis*-MR**						
Fixed effects model	0.92	0.89	0.96	0.0002	0%	0.34, 0.8455
Random effects model	0.92	0.89	0.96	0.0002		
**MR using all variants**						
Fixed effects model	0.93	0.89	0.97	0.0010	0%	0.43, 0.8054
Random effects model	0.93	0.89	0.97	0.0010		
Rectal cancer						
***cis*-MR**						
Fixed effects model	0.92	0.87	0.98	0.0083	49.8%	3.99, 0.1363
Random effects model	0.94	0.83	1.07	0.3601		
**MR using all variants**						
Fixed effects model	0.93	0.87	0.98	0.0108	40.6%	3.36, 0.1859
Random effects model	0.94	0.85	1.03	0.1590		

[a.] The meta-analysis was performed to combine the effect estimates across all three cohorts.

LPH, lactase-phlorizin hydrolase; CRC: colorectal cancer; OR: odds ratio; CI: confidence interval; MR: Mendelian Randomization.

Figure S1. Scatter plots of the IVW and MR-Egger methods investigating the effect of elevated LPH levels on CRC in the FinnGen, PLCO, and Pan-UK Biobank datasets.

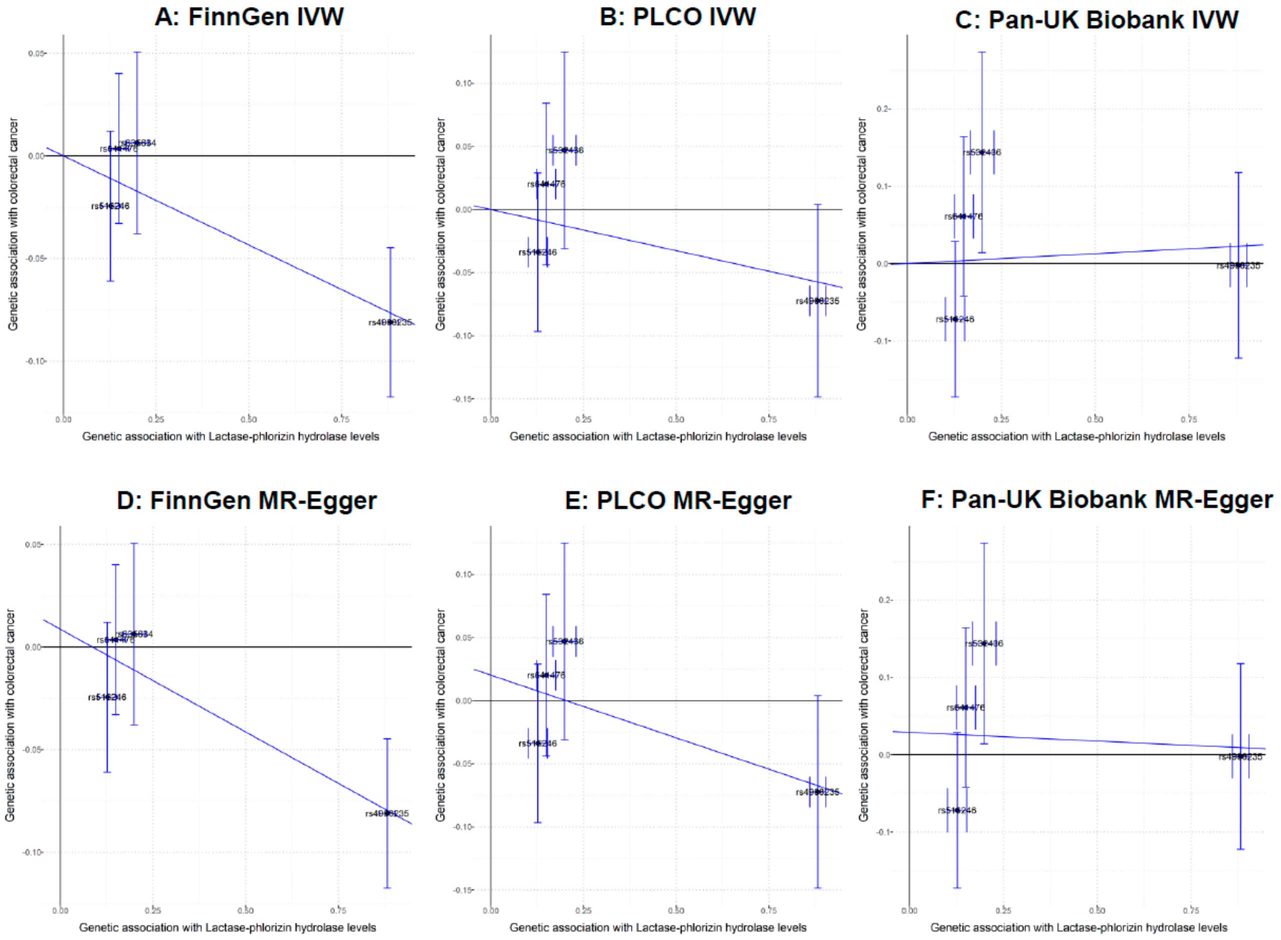

The x-axis represents the genetic association with LPH; the y-axis represents the genetic association with risk of CRC. A: FinnGen dataset using IVW method; B: PLCO dataset using IVW method; C: Pan-UK Biobank dataset using IVW method; D: FinnGen dataset using MR-Egger method; E: PLCO dataset using MR-Egger method; F: Pan-UK Biobank dataset using MR-Egger method. IVW, inverse-variance weighted; MR: Mendelian Randomization; LPH, lactase-phlorizin hydrolase; CRC, colorectal cancer.

Figure S2. Forest plots of the IVW estimates on the association between genetically predicted LPH levels and CRC risk for each genetic instrument in the FinnGen, PLCO, and Pan-UK Biobank datasets.

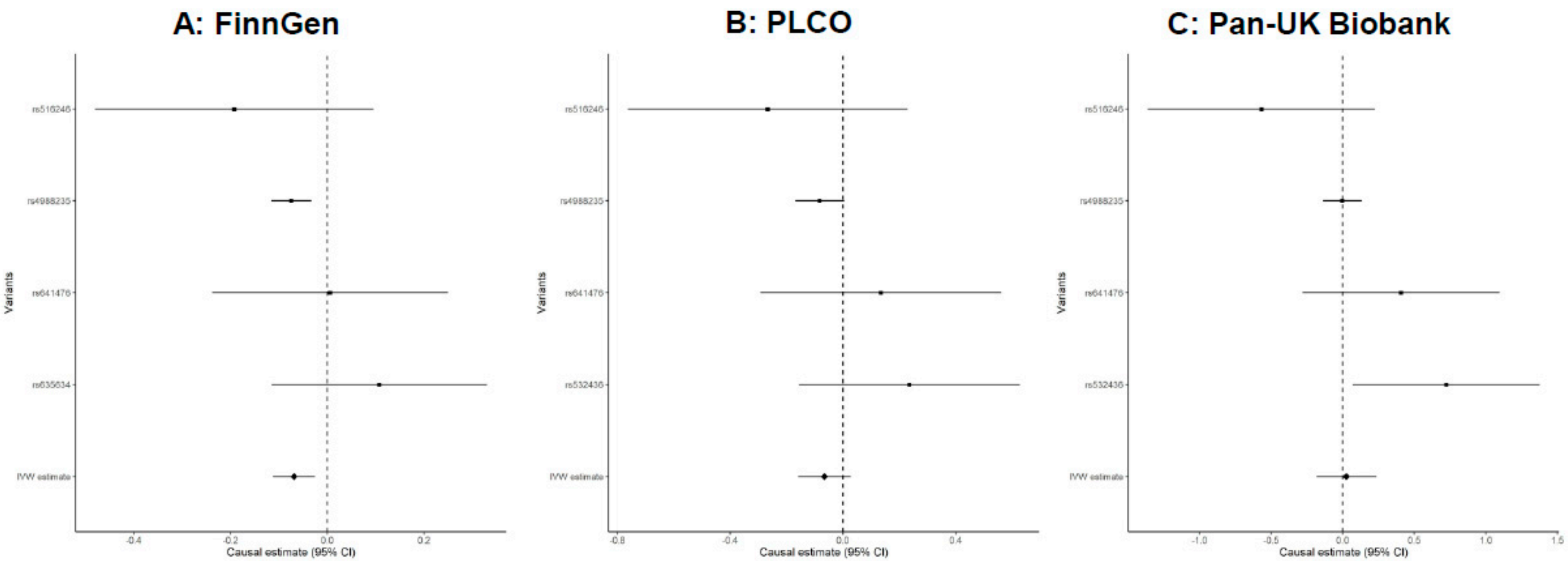

The x-axis represents the IVW causal estimate with its 95% CI; the y-axis represents genetic variant. A: FinnGen dataset; B: PLCO dataset; C: Pan-UK Biobank dataset. IVW, inverse-variance weighted; LPH, lactase-phlorizin hydrolase; CRC, colorectal cancer; CI: confidence interval.

Figure S3. Leave-one-out analyses for the MR analysis on LPH levels and CRC risk in the FinnGen, PLCO, and Pan-UK Biobank datasets.

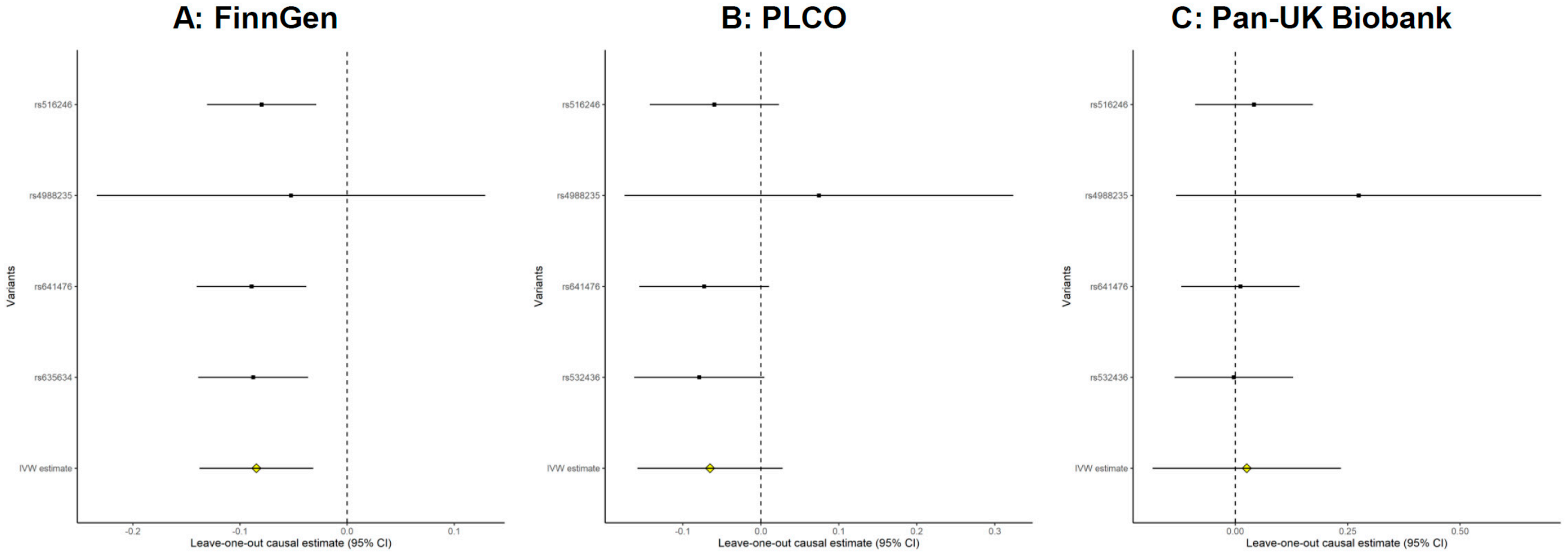

The x-axis represents the IVW estimate after removing the corresponding SNP, the Y-axis represents which genetic variant was removed from the MR analysis. MR, Mendelian Randomization; LPH, lactase-phlorizin hydrolase; CRC, colorectal cancer; IVW: inverse variance weighted; SNP, single nucleotide polymorphism; CI, confidence interval.

Figure S4. Scatter plots of the IVW and MR-Egger methods investigating the effect of elevated LPH levels on colon cancer in the FinnGen, PLCO, and Pan-UK Biobank datasets.

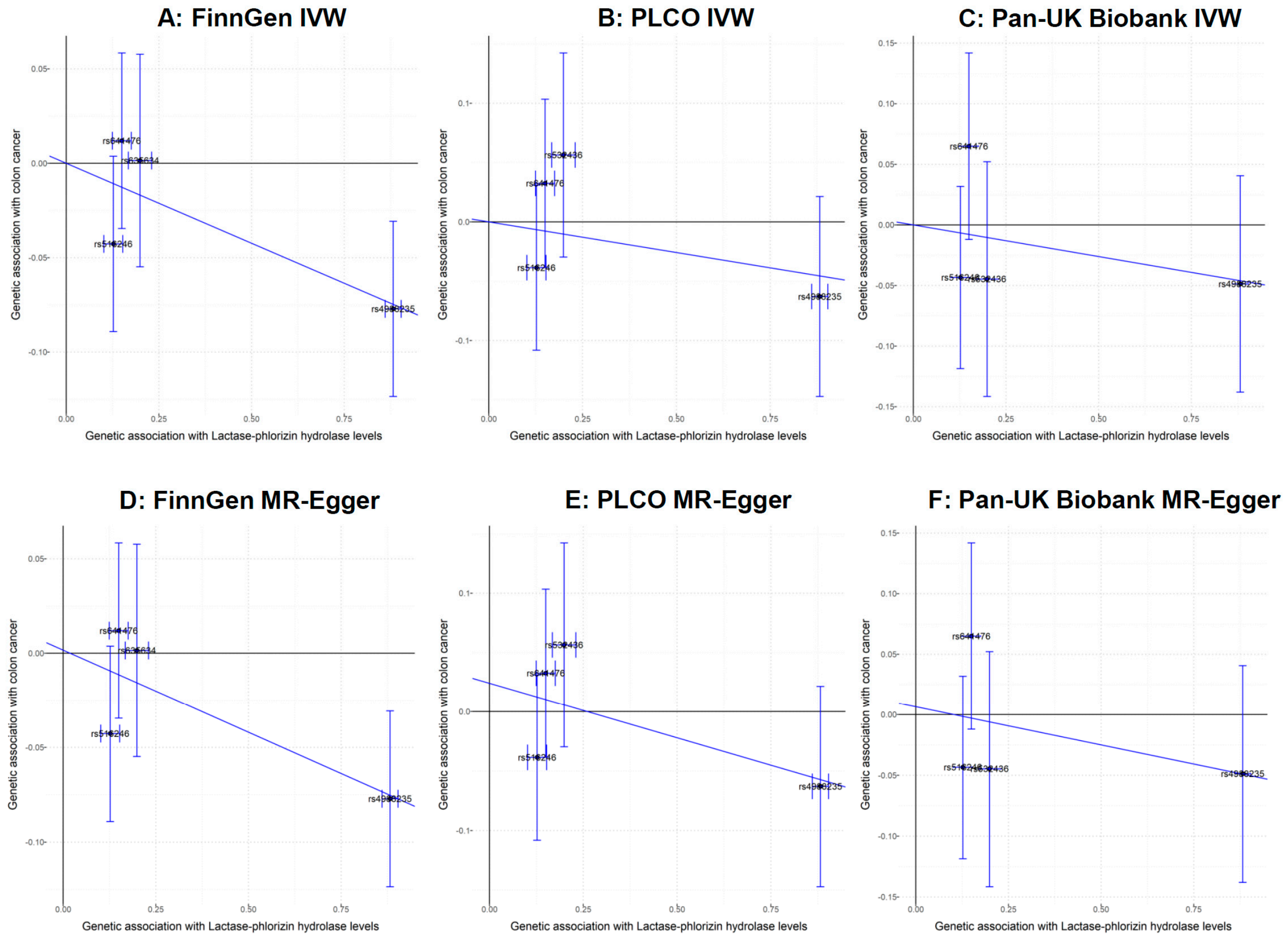

The x-axis represents the genetic association with LPH; the y-axis represents the genetic association with risk of colon cancer. A: FinnGen dataset using IVW method; B: PLCO dataset using IVW method; C: Pan-UK Biobank dataset using IVW method; D: FinnGen dataset using MR-Egger method; E: PLCO dataset using MR-Egger method; F: Pan-UK Biobank dataset using MR-Egger method. IVW, inverse-variance weighted; MR: Mendelian Randomization; LPH, lactase-phlorizin hydrolase.

Figure S5. Forest plots of the IVW estimates on the association between genetically predicted LPH levels and colon cancer risk for each genetic instrument in the FinnGen, PLCO, and Pan-UK Biobank datasets.

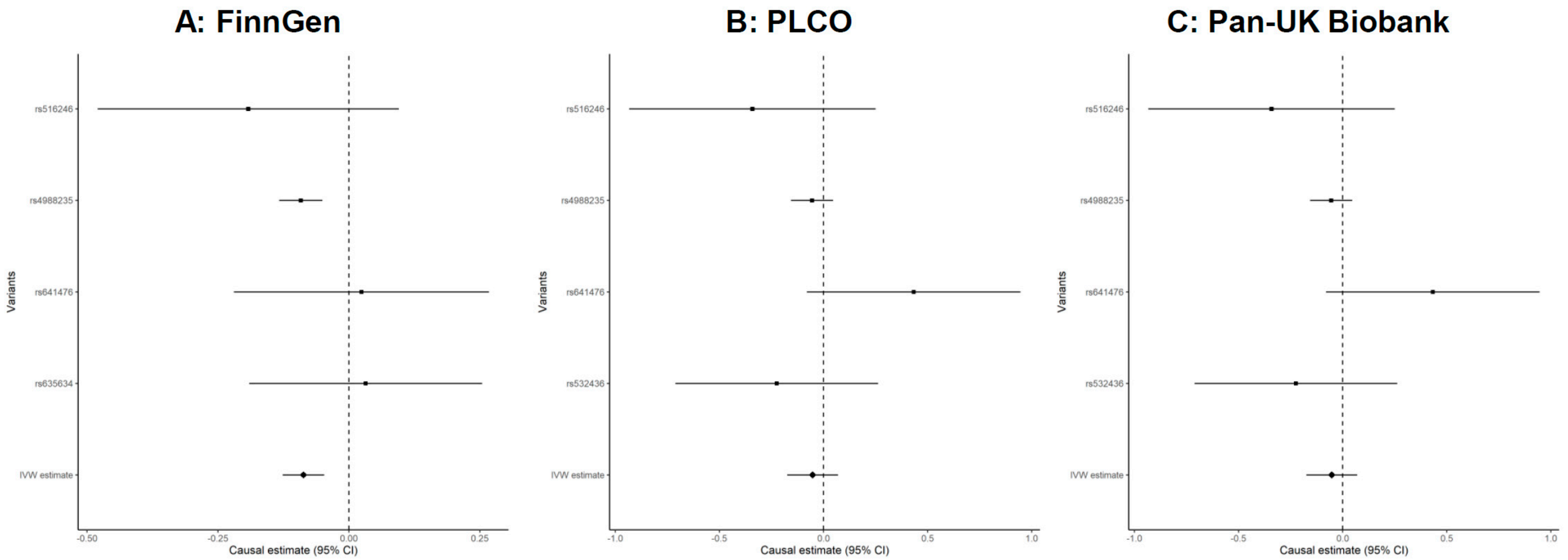

The x-axis represents the IVW causal estimate with its 95% CI; the y-axis represents genetic variant. A: FinnGen dataset; B: PLCO dataset; C: Pan-UK Biobank dataset. IVW, inverse-variance weighted; LPH, lactase-phlorizin hydrolase; CI: confidence interval.

Figure S6. Leave-one-out analyses for the MR analysis on LPH levels and colon cancer risk in the FinnGen, PLCO, and Pan-UK Biobank datasets.

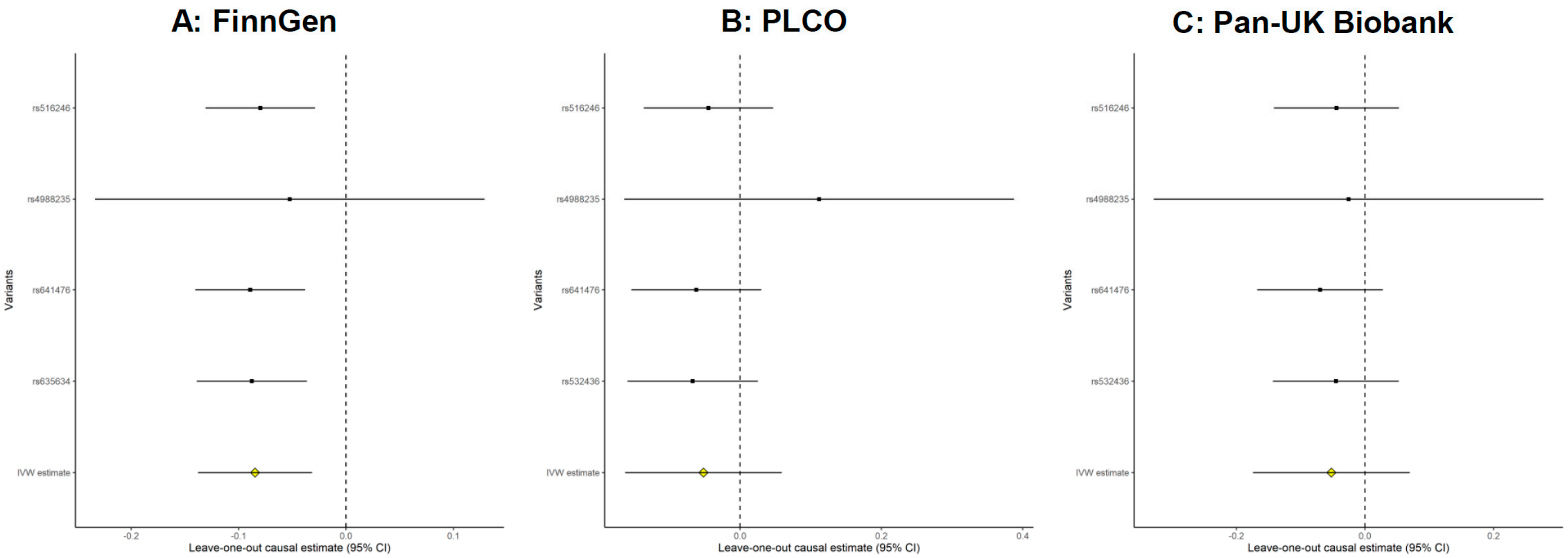

The x-axis represents the IVW estimate after removing the corresponding SNP, the Y-axis represents which genetic variant was removed from the MR analysis. MR, Mendelian Randomization; LPH, lactase-phlorizin hydrolase; IVW: inverse variance weighted; SNP, single nucleotide polymorphism; CI, confidence interval.

Figure S7. Meta-analysis results for the association of elevated LPH levels with colon cancer risk using *cis*-MR and MR using all genetic variants.

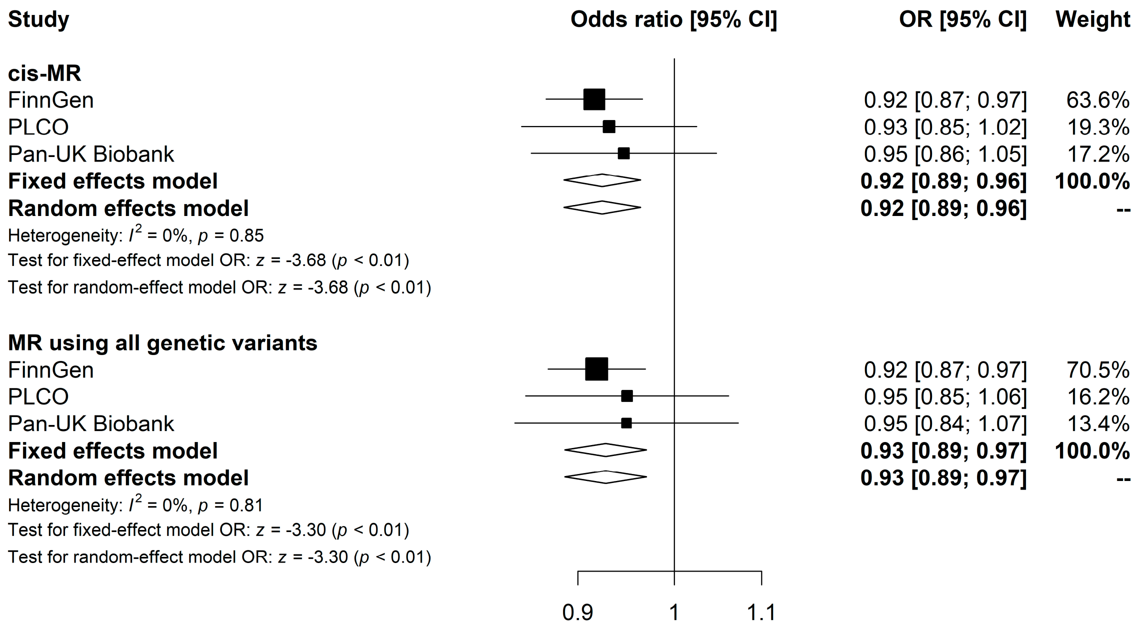

Forest plots show results from cis-MR and MR using all genetic variants. Squares represent study-specific MR estimates. Diamonds represent meta-analyzed MR estimates using fixed and random effects models. LPH, lactase-phlorizin hydrolase; MR, Mendelian Randomization.

Figure S8. Scatter plots of the IVW and MR-Egger methods investigating the effect of elevated LPH levels on rectal cancer in the FinnGen, PLCO, and Pan-UK Biobank datasets.

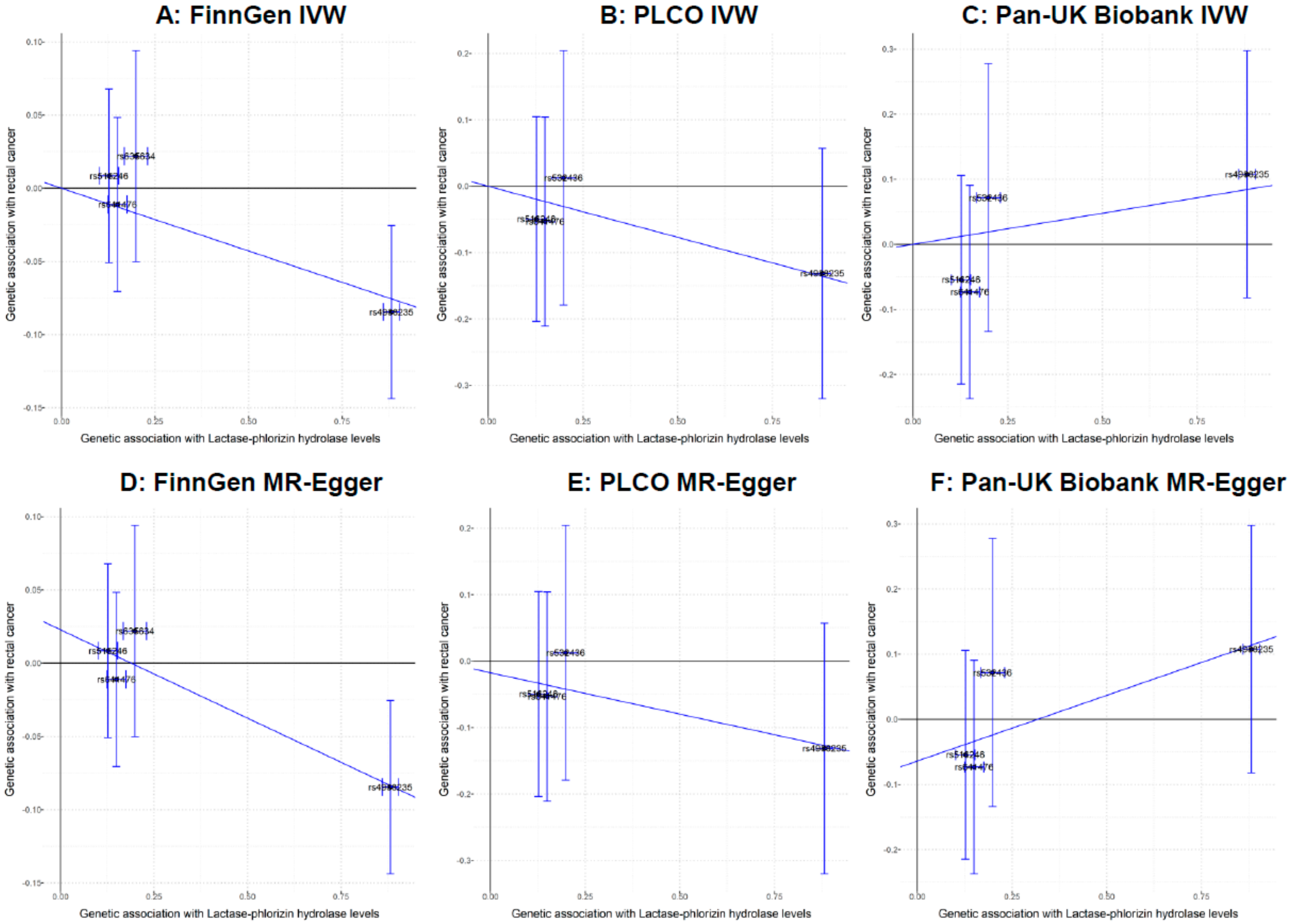

The x-axis represents the genetic association with LPH; the y-axis represents the genetic association with risk of rectal cancer. A: FinnGen dataset using IVW method; B: PLCO dataset using IVW method; C: Pan-UK Biobank dataset using IVW method; D: FinnGen dataset using MR-Egger method; E: PLCO dataset using MR-Egger method; F: Pan-UK Biobank dataset using MR-Egger method. IVW, inverse-variance weighted; MR: Mendelian Randomization; LPH, lactase-phlorizin hydrolase.

Figure S9. Forest plots of the IVW estimate on the association between genetically predicted LPH levels and rectal cancer risk for each genetic instrument in the FinnGen, PLCO, and Pan-UK Biobank datasets.

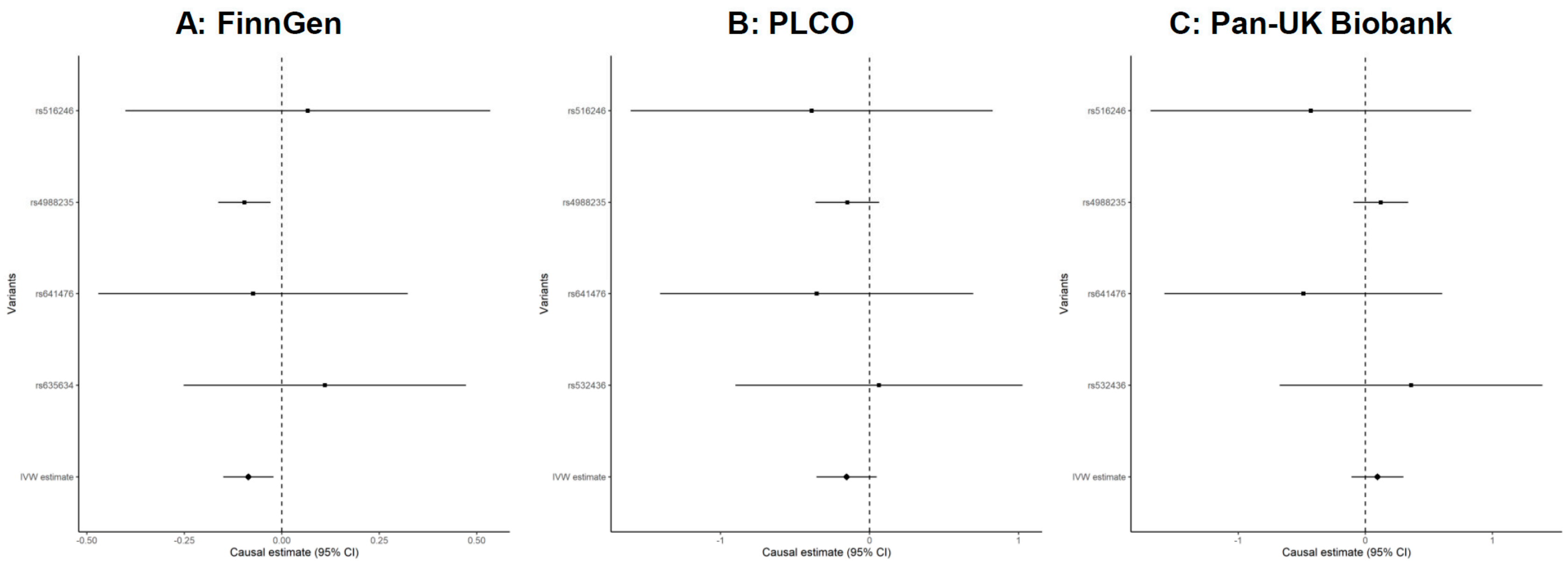

The x-axis represents the IVW causal estimate with its 95% CI; the y-axis represents genetic variant. A: FinnGen dataset; B: PLCO dataset; C: Pan-UK Biobank dataset. IVW, inverse-variance weighted; LPH, lactase-phlorizin hydrolase; CI: confidence interval.

Figure S10. Leave-one-out analyses for the MR analysis on LPH levels and rectal cancer risk in the FinnGen, PLCO, and Pan-UK Biobank datasets.

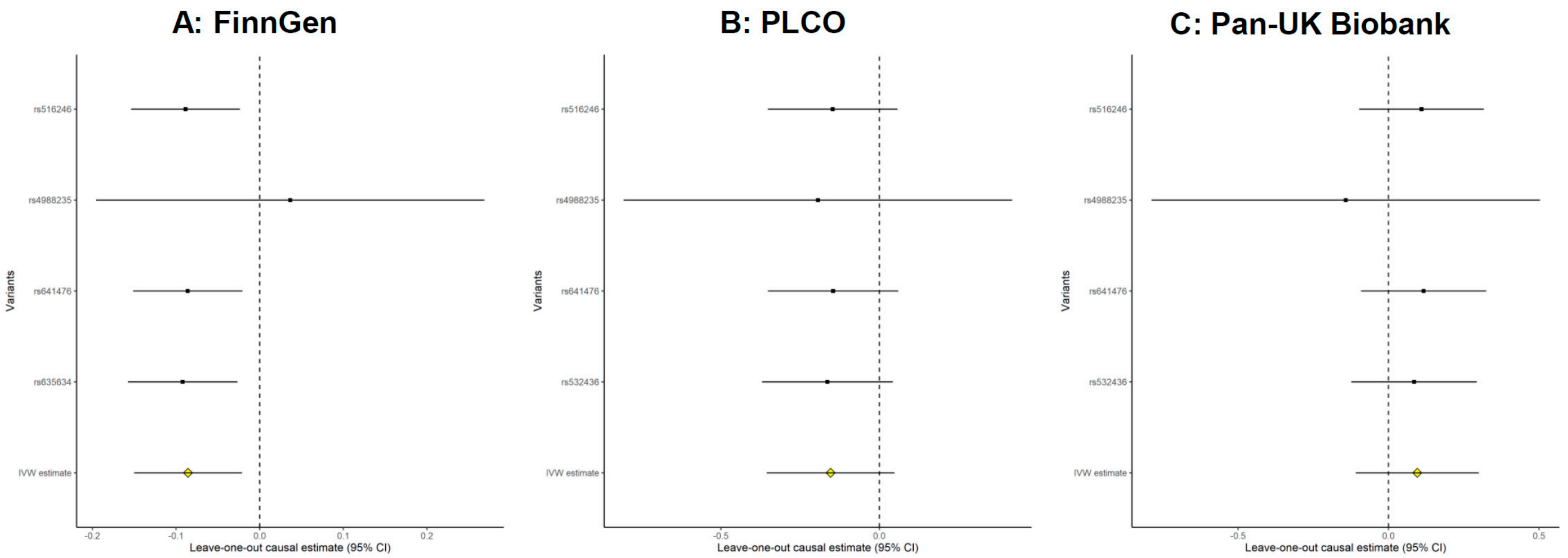

The x-axis represents the IVW estimate after removing the corresponding SNP, the Y-axis represents which genetic variant was removed from the MR analysis. MR, Mendelian Randomization; LPH, lactase-phlorizin hydrolase; CRC, colorectal cancer; IVW: inverse variance weighted; SNP, single nucleotide polymorphism; CI, confidence interval.

Figure S11. Meta-analysis results for the association of elevated LPH levels with rectal cancer risk using *cis*-MR and MR using all genetic variants.

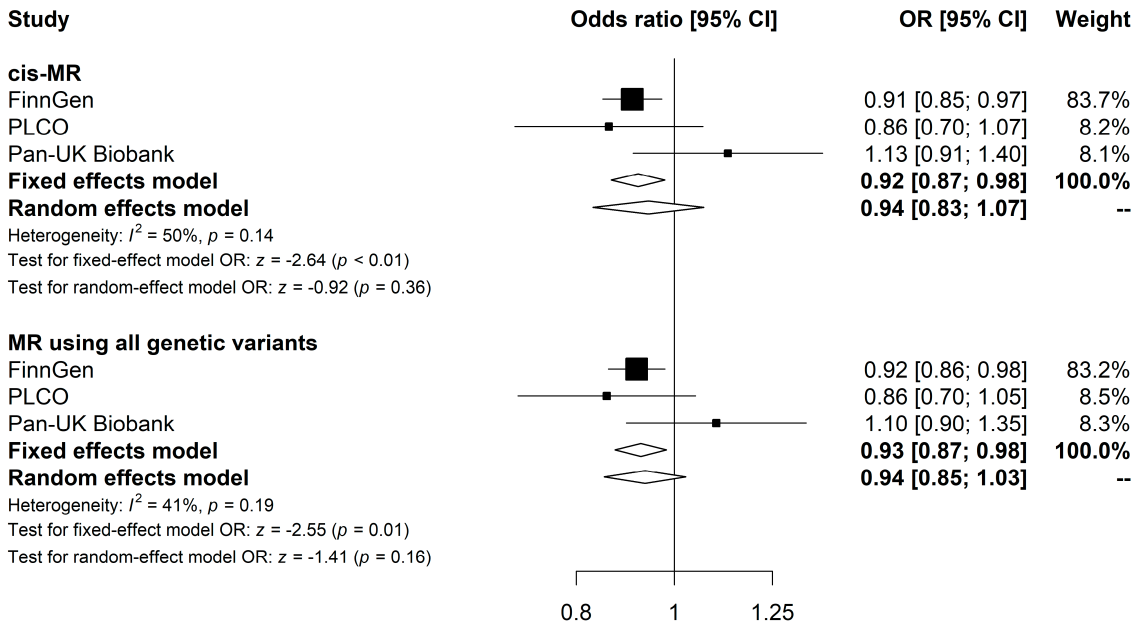

Forest plots show results from cis-MR and MR using all genetic variants. Squares represent study-specific MR estimates. Diamonds represent meta-analyzed MR estimates using fixed and random effects models. LPH, lactase-phlorizin hydrolase; MR, Mendelian Randomization.
